# Selection and characterization of ssDNA aptamers targeting tick-borne encephalitis virus particles

**DOI:** 10.1016/j.omtn.2026.103138

**Published:** 2026-10-12

**Authors:** Yuji Fujii, Tomohiro Shinozaki, Minato Hirano, Mizuki Fukuta, Taiichi Sakamoto, Kentaro Yoshii

**Affiliations:** 1 Department of Viral Ecology, Research Center for the Control and Prevention of Infectious Diseases (CCPID), Nagasaki University, Nagasaki 852-8523, Japan; 2 Experimental Animal Research Division, Research Center for the Control and Prevention of Infectious Diseases (CCPID), Nagasaki University, Nagasaki 852-8523, Japan; 3 Department of Life Science, Faculty of Advanced Engineering, Chiba Institute of Technology, Narashino, Chiba 275-0016, Japan

**Keywords:** MT: Oligonucleotides: Diagnostics and Biosensors, antiviral activity, flavivirus, ssDNA aptamer, targeted delivery, tick-borne encephalitis virus, viral diagnostics

## Abstract

Aptamers are single-stranded nucleic acids that bind to target molecules with high affinity, serving as versatile molecular tools for diagnostic and therapeutic applications. Although several aptamers against the envelope (E) protein of tick-borne encephalitis virus (TBEV) have been reported, their recognition of native viral particles remain insufficiently characterized. We identified novel ssDNA aptamers against TBEV through systematic evolution of ligands by exponential enrichment (SELEX) using a recombinant E protein that preserved the major antigenic properties of viral particles. The four major aptamer candidates showed binding activity not only to TBEV E proteins but also to TBEV-derived subviral particles. Additionally, these aptamers recognized TBEV-infected cells and retained their binding activity under serum conditions mimicking the *in vivo* environment. Some aptamers also exhibited inhibitory effects against TBEV infection *in vitro*. Furthermore, sequence truncation yielded optimized variants that retained high target affinity, demonstrated enhanced serum stability over 24 h, and neutralized viral infection. These findings provide crucial functional validation of ssDNA aptamers targeting TBEV particles and support their development as antibody-independent platforms for diagnostic, antiviral, and targeted delivery strategies for TBEV and related flaviviruses.

## Graphical abstract

**Figure:**
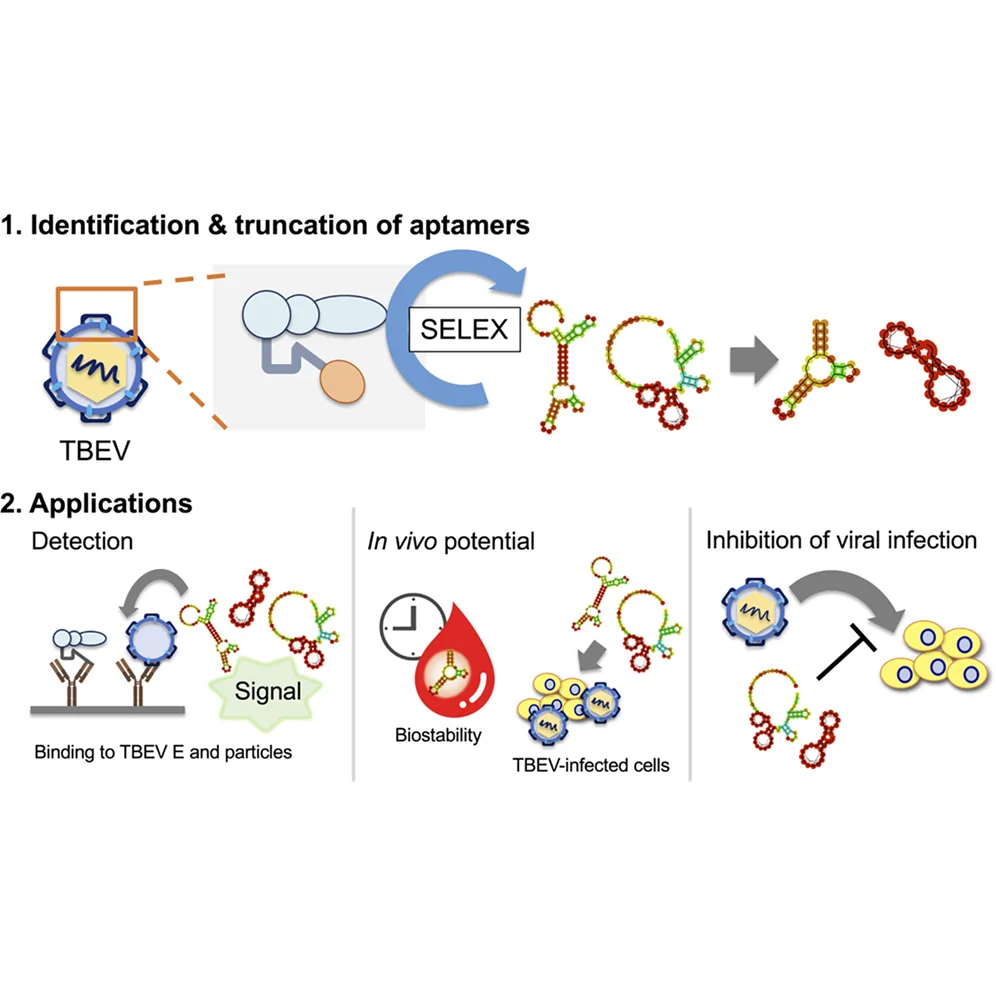

## Teaser

Tick-borne encephalitis virus (TBEV) causes severe neurological disease, highlighting the need for targeted diagnostic and therapeutic strategies. Yoshii and colleagues identified ssDNA aptamers that recognize TBEV particles and infected cells, retain activity under serum conditions, and can be optimized for stability, providing a foundation for aptamer-based diagnostics and targeted delivery.

## Introduction

Aptamers are single-stranded nucleic acids (DNA or RNA), typically 10–100 nucleotides (nt) in length, that fold into defined three-dimensional structures. These structures enable aptamers to bind to specific target molecules with high affinity, similarly to antibodies. Consequently, aptamers have been widely investigated as alternatives to antibodies, particularly in ophthalmology and *in vitro* diagnostics.^1^^,^^2^ Furthermore, compared to antibodies, aptamers possess several advantages, including ease of synthesis, low immunogenicity, and amenability to chemical modification. Owing to these characteristics, their applications in bioimaging and drug delivery systems (DDS) are increasingly recognized as versatile tools for both diagnostic and therapeutic applications.^3^^,^^4^^,^^5^

Tick-borne encephalitis virus (TBEV), a member of the *Flaviviridae* family, causes tick-borne encephalitis, which can lead to severe neurological complications.^6^^,^^7^ Following infection, TBEV exhibits complex pathogenesis involving various cell types and tissues.^8^ This process is divided into three stages: initial replication at the inoculation site, systemic dissemination via viremia, and subsequent invasion of the central nervous system (CNS).^9^ Moreover, viral replication within the CNS, where selective drug delivery remains challenging, further complicates lesion-selective treatment of TBEV infection. Therefore, studies on diagnostic strategies and DDS targeting TBEV serve as useful models for other viral infections.

The envelope (E) protein of TBEV is a critical structural component of the virion, and several aptamers targeting this protein have been reported. However, most previous studies have focused on binding analyses of recombinant E proteins or isolated antigens,^10^^,^^11^ whose relationship to the antigenic properties of native TBEV particles has not been fully characterized. Thus, whether these aptamers bind to native TBEV particles remains largely unverified.^11^^,^^12^ Furthermore, little is known about the extent to which anti-TBEV aptamers interact with virions or whether they possess properties suitable for *in vivo* administration. These limitations indicate a significant lack of foundational data required to validate aptamer-based strategies not only for *in vitro* diagnostics but also for targeted delivery and therapeutic applications.

In this study, we identified novel ssDNA aptamers using a recombinant E protein that retained the major antigenic epitopes of native TBEV particles, and characterized their biochemical and functional properties. Specifically, we evaluated the binding affinity and specificity of the identified aptamers toward TBEV particles, antigens, and infected cells, and assessed their applicability in an aptamer-based *in vitro* diagnostic system. Furthermore, to obtain preliminary information for the *in vivo* application of TBEV-specific ssDNA aptamers, we examined their biological stability, ability to recognize TBEV-infected cells, and capacity to inhibit viral infection. We also performed sequence truncation analyses to optimize aptamer length while retaining functional activity.

## Results

### Identification of TBEV-specific ssDNA aptamers

The systematic evolution of ligands by exponential enrichment (SELEX) method was used to generate aptamers that bind to target molecules through iterative cycles of selection, purification, and amplification. The choice of target molecule is critical for obtaining specific aptamers. In the present study, TBEV E-Fc was used as the SELEX target (Figure 1C). The E-Fc protein is a fusion protein in which the rabbit IgG Fc region is fused to the transmembrane region of the TBEV E protein. As previously reported, this protein exhibits antigenic properties similar to those of the E protein, which constitutes the TBEV virion.^13^ The Fc region serves as a tag to facilitate purification during SELEX.

**Figure 1:**
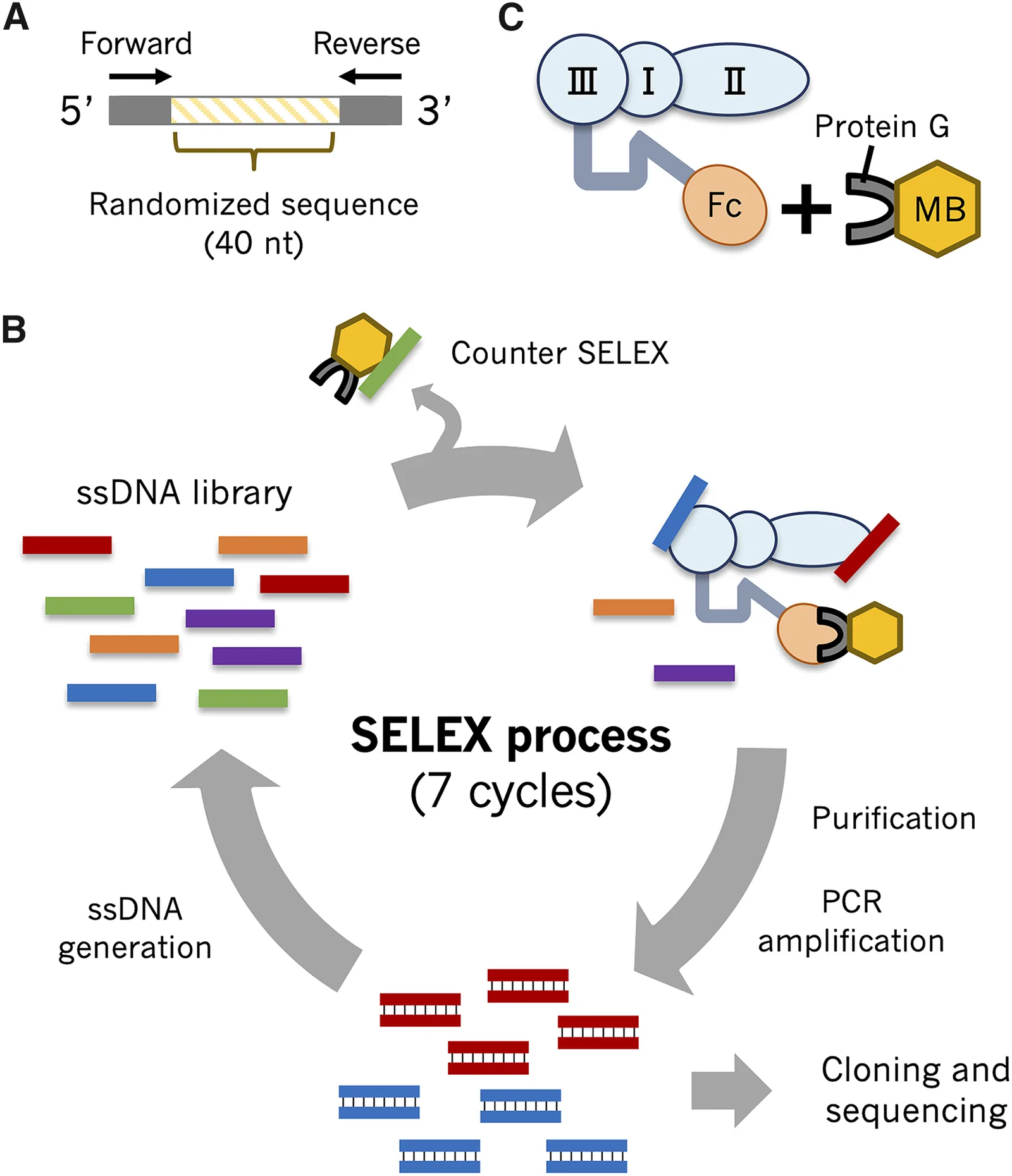
Development of ssDNA aptamers against TBEV E protein (A) Composition of the ssDNA aptamer library. The library consisted of 81-nucleotide sequences containing a central 40-nucleotide randomized region flanked by primer-binding sites. (B) Schematic representation of the systematic evolution of ligands by exponential enrichment (SELEX) protocol. (C) Preparation of the target for aptamers. E-Fc (domains I, II, and III), a recombinant protein comprising the C-terminal region of the TBEV E protein fused to rabbit IgG Fc, was conjugated to protein G-coated magnetic beads (MB).

After seven rounds of aptamer selection against E-Fc, nucleotide sequences were obtained from 55 clones. These sequences were distinct from previously reported aptamers (TBEV-2F, TBEV-2R, and TBEV-8R) that bound to the TBEV E protein.^10^ Phylogenetic tree analysis classified the sequences obtained in this study into ten clusters (Figure 2A). Among these clusters, the four most frequently identified sequences, aptE-Fc/3-1, aptE-Fc/3-2, aptE-Fc/3-3, and aptE-Fc/3-4, were selected for subsequent analyses (Table 1).

**Figure 2:**
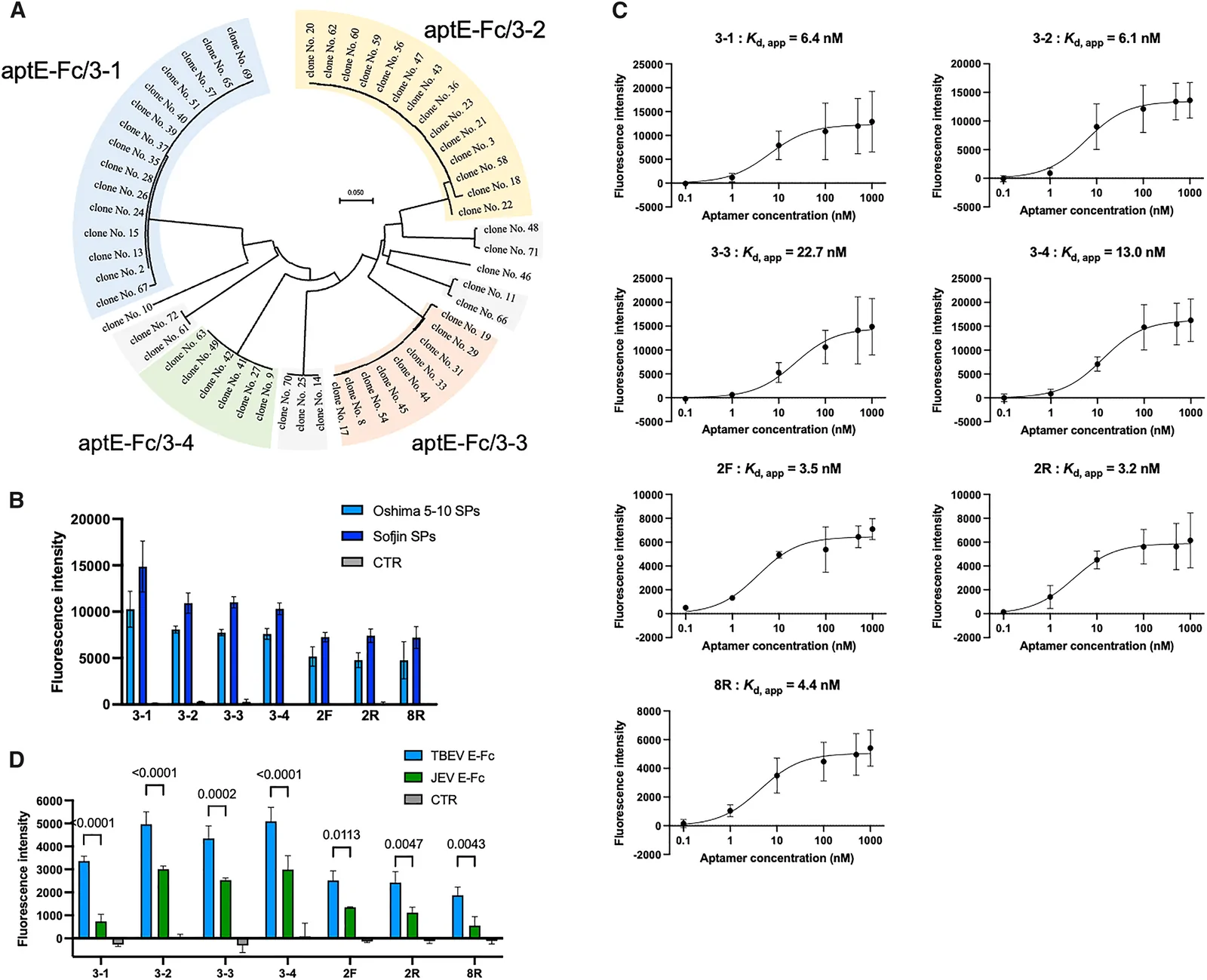
Classification and binding affinity of aptamer candidates identified in this study (A) Phylogenetic analysis of aptamer candidate sequences. Sequences with ≥95% homology are highlighted with background shading. The four most frequent sequences aptE-Fc/3-1, aptE-Fc/3-2, aptE-Fc/3-3, and aptE-Fc/3-4 are indicated in blue, yellow, orange, and green, respectively. (B) Screening of binding affinity using TBEV subviral particles (SPs). Binding of each aptamer (100 nM) was evaluated using SPs derived from two TBEV strains, Oshima 5-10 and Sofjin. (C) Binding curves of aptamers against SPs derived from the Oshima 5-10 strain. CTR represents the negative control without SPs. Apparent dissociation constants (*K*_d, app_) were calculated using GraphPad Prism 9.5.1 by a nonlinear regression fit based on a one-site binding model. (D) Evaluation of aptamer specificity. Binding affinities to TBEV E-Fc were compared with those to Japanese encephalitis virus (JEV) E-Fc, which exhibits antigenic similarity with TBEV. CTR represents the negative control without E-Fc. Statistical analysis was performed with ANOVA. Data are presented as mean ± standard deviation.

**Table 1:** The ssDNA aptamers used in this study

Aptamer	Sequences^a^	Reference
aptE-Fc/3–1	agtatacgtattacctgcagcTAATTTCGTCGATGTATGTCGAGTTCCCTTGGTAAATTAGcgatatctcggagatcttgc	this study
aptE-Fc/3–2	agtatacgtattacctgcagcATGGTGGAGGAGGGGGTTGGGAGGGGGGGTCGCGTTTCAAcgatatctcggagatcttgc	this study
aptE-Fc/3–3	agtatacgtattacctgcagcTGGATGGTGGTGGGGGAGGGTGGGTTGGGTTTTTTATCCGcgatatctcggagatcttgc	this study
aptE-Fc/3–4	agtatacgtattacctgcagcGGGGGGGTTGGGTGGGCTTGCGGATTATTATCCGCTAACTcgatatctcggagatcttgc	this study
aptE-Fc/3–1_37	GCGTTCGTCGATGTATGTCGAGTTCCCTTGGTAACGC	this study
aptE-Fc/3–2_32	GGTGGAGGAGGGGGTTGGGAGGGGGGGTCGCC	this study
TBEV-2F	ATACGGGAGCCAACACCACCGCAGGAGTCCATCAGGGGTTGGCAGTCAGCGCTCAGAGCAGGTGTGACGGAT	Bruno et al., 2012
TBEV-2R	ATCCGTCACACCTGCTCTGAGCGCTGACTGCCAACCCCTGATGGACTCCTGCGGTGGTGTTGGCTCCCGTAT	Bruno et al., 2012
TBEV-8R	ATCCGTCACACCTGCTCTACATCACATCTGACTGAGTCCATGGCGCTCTGTGTGTGGTGTTGGCTCCCGTAT	Bruno et al., 2012

a Primer binding sequences are shown in lowercase.

To compare the binding of the selected aptamers to TBEV particles, binding analyses were performed using subviral particles (SPs). SPs are composed of prM and E proteins and do not contain the Fc region. As previously reported, SPs exhibit antigenic properties comparable to those of infectious TBEV particles.^14^^,^^15^ A screening assay with 100 nM of each FAM-labeled aptamer revealed detectable fluorescence intensity in the presence of Oshima 5-10 and Sofjin SPs (Figure 2B). In contrast, all aptamers exhibited minimal reactivity in the absence of SPs. The apparent dissociation constant (*K*_d, app_) value for each aptamer toward the Oshima 5-10 SPs was calculated using a one-site binding model, as described previously.^16^ The *K*_d, app_ values of the aptE-Fc/3-1 and aptE-Fc/3-2 were 6.4 nM (95% confidence interval [CI]: 1.2-33.1 nM) and 6.1 nM (95% CI: 2.3-15.8 nM), respectively. These *K*_d, app_ values were comparable to those of the previously reported aptamers (TBEV-2F, TBEV-2R, and TBEV-8R; 3.5 nM [95% CI: 1.6–7.6 nM], 3.2 nM [95% CI: 1.0–9.4 nM] and 4.4 nM [95% CI: 1.5–12.1 nM], respectively) (Figure 2C). In contrast, the *K*_d, app_ values of aptE-Fc/3-3 and aptE-Fc/3-4 (22.7 nM [95% CI: 5.0–118.3 nM] and 13.0 nM [95% CI: 5.2–32.8 nM], respectively) were higher than those of the other aptamers (Figure 2D). These results indicated that the novel aptamers aptE-Fc/3-1 and aptE-Fc/3-2 exhibited high binding affinity to TBEV viral particles.

As TBEV exhibits antigenic similarity to Japanese encephalitis virus (JEV), another neurotropic flavivirus, some anti-TBEV antibodies cross-react with JEV.^17^ To evaluate the specificity of each aptamer, binding was compared using plates immobilized with equal amounts (50 ng) of E-Fc derived from TBEV and JEV (Figure 2D). Binding assays using 100 nM of each FAM-modified aptamer revealed that aptE-Fc/3-1, aptE-Fc/3-2, aptE-Fc/3-3, and aptE-Fc/3-4 exhibited significantly higher fluorescence intensities in the presence of TBEV E-Fc than JEV E-Fc. This indicates that all aptamers obtained in this study recognized the TBEV E protein and exhibited preferential binding to TBEV over the closely related JEV E protein.

### Truncation of aptamers

The predicted secondary structures of aptE-Fc/3-1 and aptE-Fc/3-2 are shown in Figures 3A and 3B, respectively. AptE-Fc/3-1 was predicted to contain a stem-loop structure in the region flanked by the primer-binding sites. AptE-Fc/3-2 is composed of guanine-rich sequences, and secondary structure prediction suggests the potential formation of G-quadruplex structures.^18^^,^^19^ Based on these structural models, truncated variants of each aptamer, aptE-Fc/3-1_37 and aptE-Fc/3-2_32, were designed (Figures 3A and 3B). Furthermore, we measured the nuclear magnetic resonance (NMR) spectra of aptE-Fc/3-2_32 to examine its G-quadruplex formation (Figure S1). Although the signals could not be assigned due to their broadness, imino proton signals were observed in the 10–12 ppm range; these signals were very broad in the presence of Na^+^, whereas some sharp signals were observed in the presence of K^+^. When compared with previous reports,^20^^,^^21^^,^^22^ these results indicate that aptE-Fc/3-2_32 forms a G-quadruplex structure.

**Figure 3:**
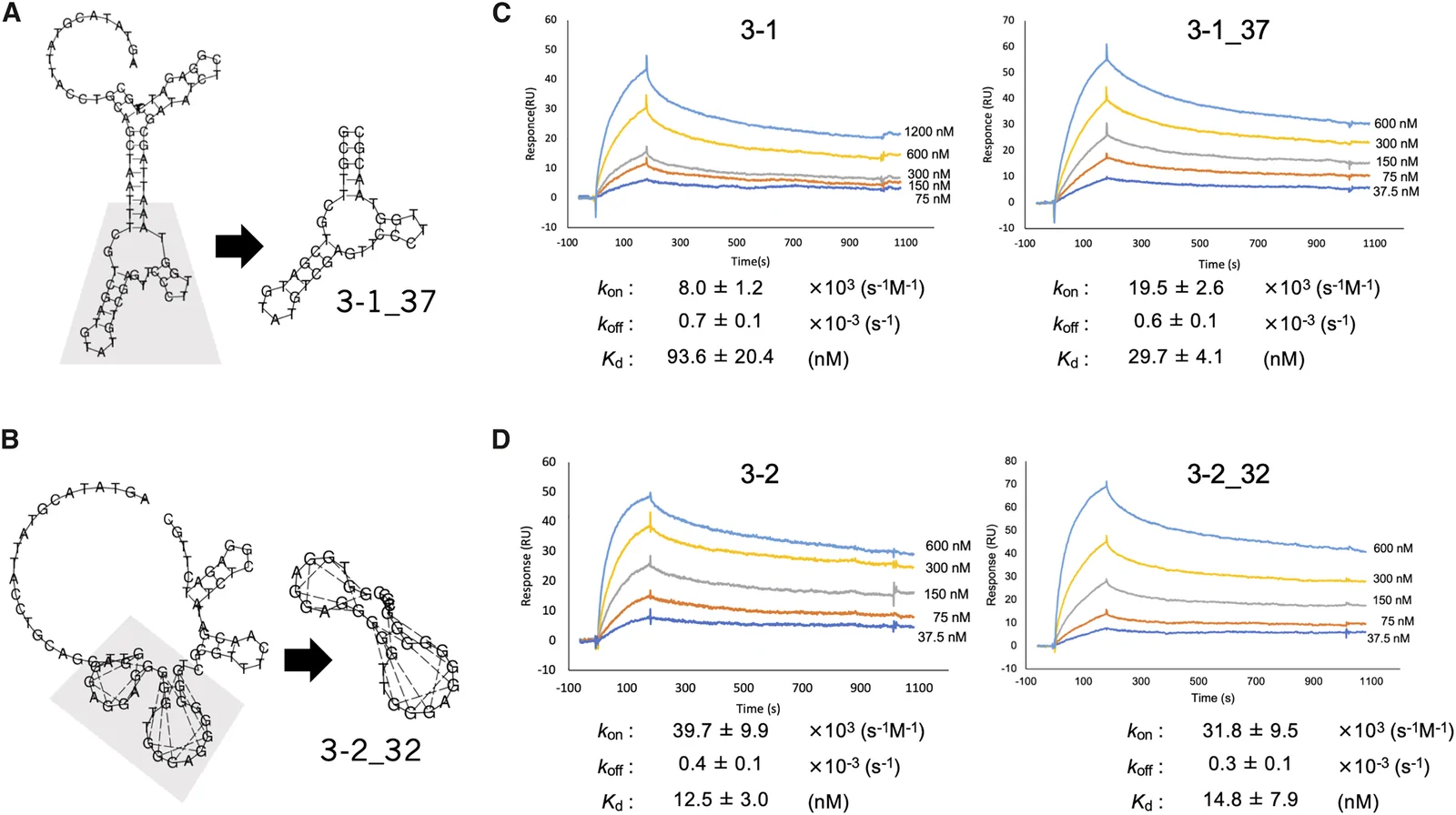
Truncation of aptamers (A and B) Predicted DNA secondary structure of the full-length and truncated forms of aptE-Fc/3-1 (A) and aptE-Fc/3-2 (B) using the RNAfold web server (http://rna.tbi.univie.ac.at/cgi-bin/RNAWebSuite/RNAfold.cgi). Dashed lines indicate regions predicted to form G-quadruplex (G4) structures. (C and D) SPR sensorgrams of the interaction between TBEV E-Fc and aptamers.

Surface plasmon resonance (SPR) analysis using TBEV E-Fc revealed that the association rate constant (*k*_on_) of the truncated variant aptE-Fc/3-1_37 (19.5 × 10^3^ s^−1^M^−1^) was higher than that of the original aptamer (8.0 × 10^3^ s^−1^M^−1^). This increase in the association rate resulted in a lower *K*_d_ value for aptE-Fc/3-1_37 (29.7 nM) than for the original aptE-Fc/3-1 (93.6 nM) (Figure 3C). These results suggest that truncation stabilized the active structure of aptE-Fc/3-1_37, thereby accelerating binding. The *K*_d_ value of aptE-Fc/3-2_32 (14.8 nM) was comparable to that of the original aptamer aptE-Fc/3-2 (12.5 nM) (Figure 3D), indicating that this truncated variant retained sequences critical for binding affinity from the original aptamer.

### Applications of aptamers for diagnostic assays

To evaluate whether the newly identified aptamers were applicable to TBEV detection, validation assays were performed using surfactant-treated TBEV. Specifically, 1.25 × 10^5^ plaque-forming units (PFU) of Triton X-100-treated Oshima 5-10 strain was immobilized on a microplate with anti-TBEV E antibodies and detected using biotin-modified aptamers (800 nM) and HRP-conjugated streptavidin. AptE-Fc/3-1 and aptE-Fc/3-2 showed significantly higher optical density (OD) values for TBEV than for the negative control (Figure 4A). Furthermore, the truncated aptamers, aptE-Fc/3-1_37 and aptE-Fc/3-2_32, showed increased OD values in the presence of TBEV. In contrast, no increase in the OD values was observed in the wells that reacted with the previously reported aptamer, TBEV 2R. These results indicated that aptE-Fc/3-1 and aptE-Fc/3-2, as well as their truncated variants, bind to TBEV.

**Figure 4:**
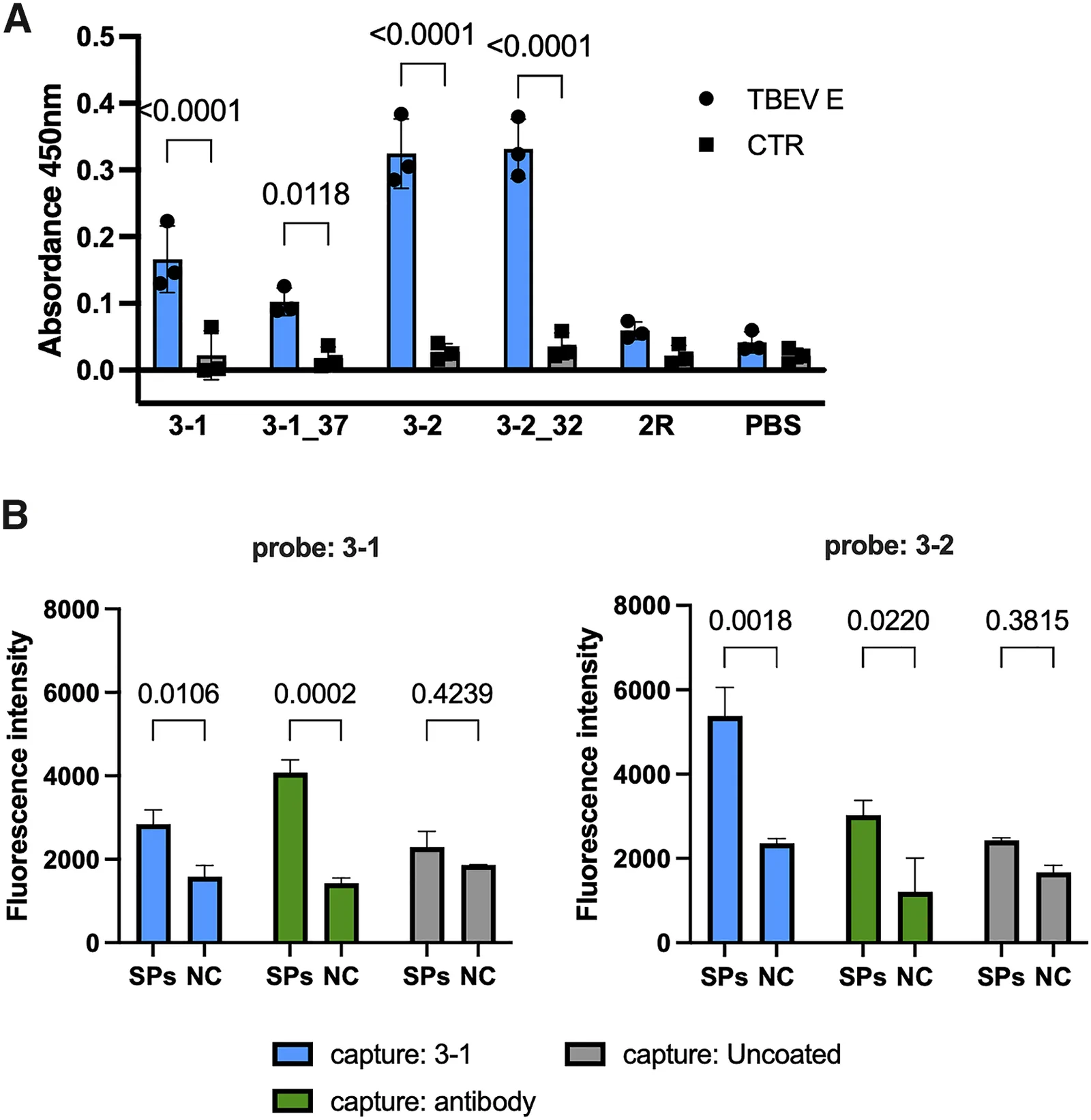
Evaluation of an aptamer-based diagnostic assay (A) Detection of TBEV following surfactant treatment. The inactivated Oshima 5-10 strain (1.25 × 10^5^ PFU) was immobilized on a microplate coated with anti-TBEV E antibodies and detected using a biotinylated aptamer (800 nM) and HRP-conjugated streptavidin. CTR represents the negative control without TBEV. (B) Antibody-independent detection of TBEV. Subviral particles (SPs) were captured on a microplate coated with aptE-Fc/3-1 (500 nM) or anti-TBEV E antibodies, or on uncoated microplates, and detected using a FAM-labeled aptamer as a probe. NC represents the negative control without SPs. Statistical analysis was performed with ANOVA. Data are presented as mean ± standard deviation.

To establish an antibody-independent diagnostic system, aptE-Fc/3-1 was used as the capture aptamer and aptE-Fc/3-1 or aptE-Fc/3-2 as the probe aptamer. For both aptamer pairs, fluorescent signals were detected in the presence of SPs (Figure 4B). The results obtained with these aptamer pairs are consistent with those observed in the system using anti-TBEV E antibodies as the capture agent.

### Biostability of aptamers

To evaluate whether the aptamers retained their binding activity under physiological conditions, their stability was examined in serum (Figure 5A). The residual binding activities of the ssDNA aptamers aptE-Fc/3-1 and aptE-Fc/3-2 after 2 h were more than 70%. In contrast, both aptamers exhibited substantial loss of activity after 24 h. The binding affinity of the truncated aptamer aptE-Fc/3-1_37 was significantly lower than that of aptE-Fc/3-1 at the 3 h time point, though its overall kinetic baseline profile mirrored the parental sequence. The binding activity of aptE-Fc/3-2_32 remained largely unchanged even after 24 h in mouse serum.

**Figure 5:**
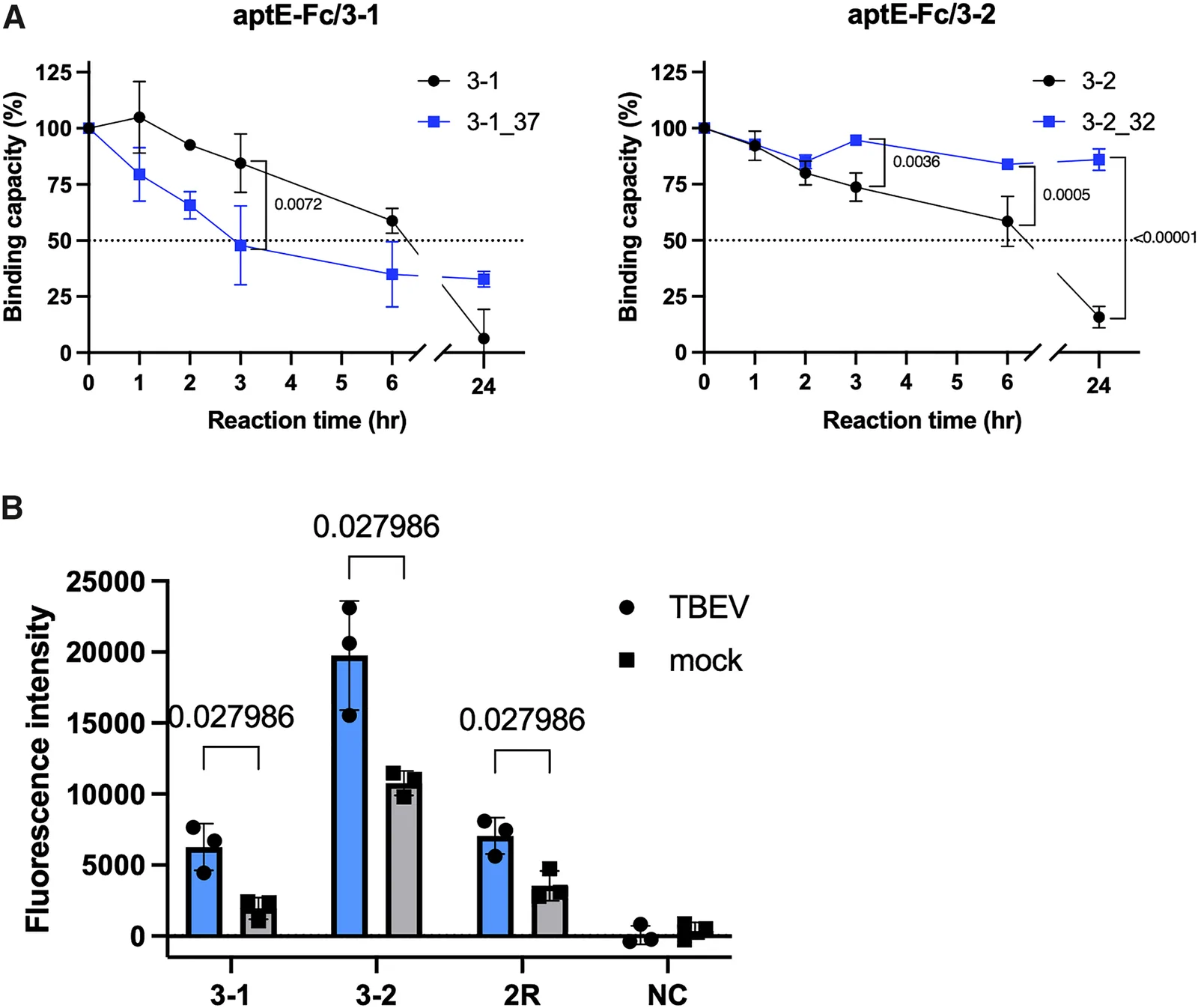
Biological stability of aptamers and their binding affinity to TBEV-infected cells (A) Dynamics of the binding ability of aptamers in the presence of mouse serum. Aptamers were incubated in 50% mouse serum for the indicated time periods, and their binding activity to subviral particles (SPs) was subsequently evaluated. (B) Binding of aptamers to TBEV-infected cells. The Alexa Flour 647-labeled aptamer was incubated with TBEV-infected cells 2 days post-infection, and binding was assessed. Mock indicates uninfected cells. Statistical analysis was performed with ANOVA. Data are presented as mean ± standard deviation.

### Binding of aptamers to TBEV-infected cells

To evaluate whether the aptamers identified in this study were suitable for detecting infected cells, fluorescently labeled aptamers were reacted with TBEV-infected cells. AptE-Fc/3-1, aptE-Fc/3-2, and TBEV 2R exhibited significantly higher fluorescence intensities in TBEV-infected cells than in uninfected cells (Figure 5B; Figure S2). These results indicated that each aptamer selectively bound to the infected cells.

### Inhibition of viral infection by aptamers

To evaluate the inhibitory effects of the aptamers on viral infection, a modified plaque reduction neutralization test (PRNT) was performed (Figure 6A). Plaque counts of the JEV JaOArS982 strain in the presence of each aptamer (1 μM) were comparable to those in the ssDNA control (1 μM of library). In contrast, the plaque counts of the TBEV Oshima 5-10 strain were reduced in the presence of aptE-Fc/3-2, aptE-Fc/3-3, aptE-Fc/3-4, and TBEV-2R. Furthermore, aptE-Fc/3-2 showed a significant reduction in plaque counts for Langat virus (LGTV) strain TP21, another member of the TBEV serocomplex.

**Figure 6:**
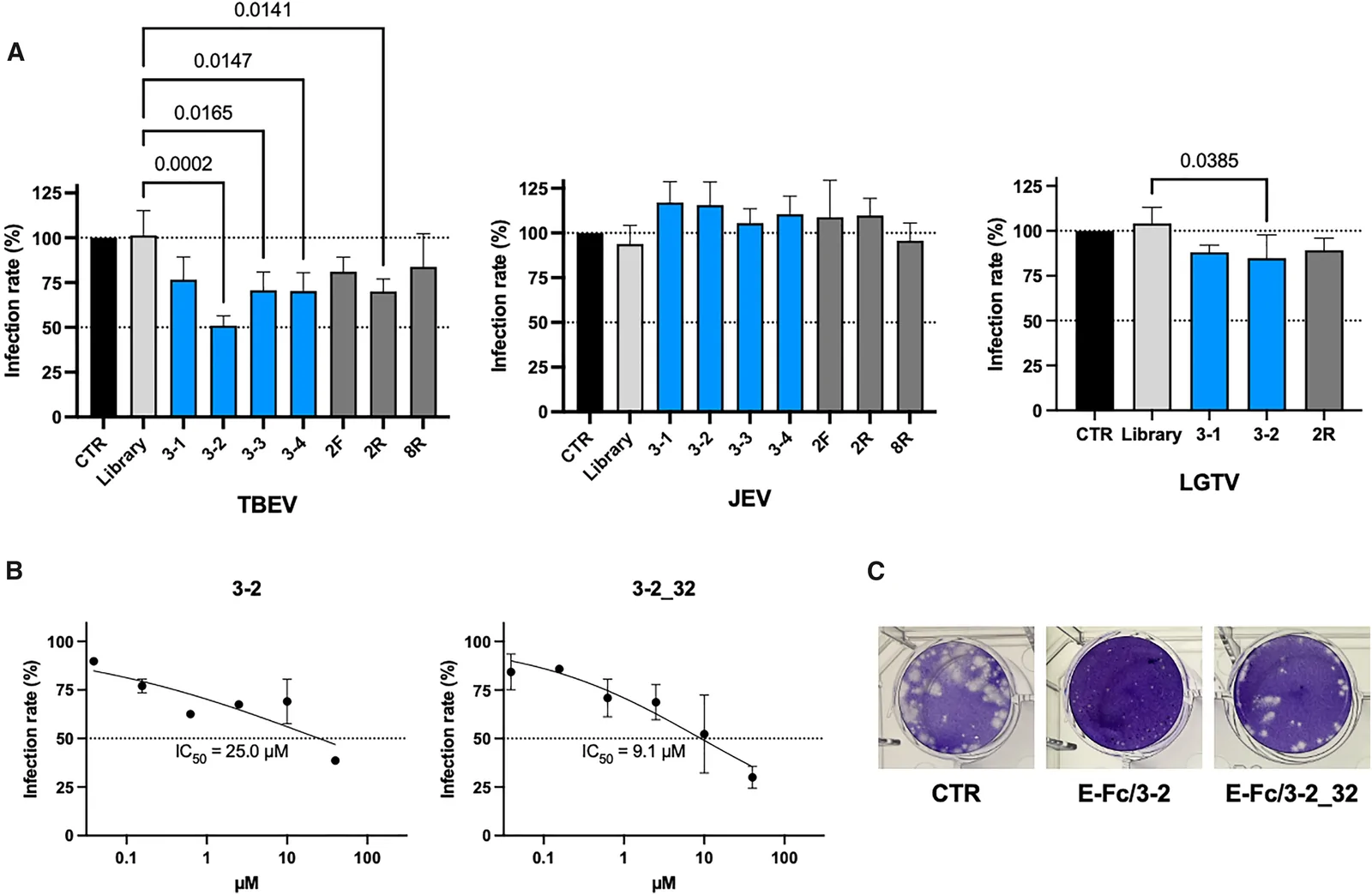
Inhibition of viral infection by aptamer (A) Screening of the inhibitory effects of aptamers on viral infection. Newly identified aptamers aptE-Fc/3-1, aptE-Fc/3-2, aptE-Fc/3-3, and aptE-Fc/3-4 (final concentration 1 μM) were evaluated against TBEV, Japanese encephalitis virus (JEV), and Langat virus (LGTV) using a plaque assay. Previously reported aptamers 2F, 2R, and 8R were included for comparison. Evaluation against the LGTV was performed only for aptE-Fc/3-1, aptE-Fc/3-2, and 2R. Plaque counts were normalized to the negative control (PBS only: CTR). Statistical analysis was performed with ANOVA, with comparisons made against a random sequence control (Library). (B) Inhibition of viral infection by aptamers at varying concentrations. The half-maximal inhibitory concentration (IC_50_) was calculated using a nonlinear regression model. Data are presented as mean ± standard deviation. (C) Representative plaque images of TBEV mixed with aptamers aptE-Fc/3-2 and aptE-Fc/3-2_32 at a final concentration of 40 μM.

Next, we investigated whether the truncated aptamer aptE-Fc/3-2_32 retained its ability to inhibit viral infection. Serial dilution experiments revealed that aptE-Fc/3-2 inhibited TBEV infection in a concentration-dependent manner, with a half-maximal inhibitory concentration (IC_50_) of 25.0 μM (95% CI: 8.2 to 239.5 μM). Similarly, aptE-Fc/3-2_32 suppressed TBEV infection and exhibited an IC_50_ of 9.1 μM (95% CI: 3.2 to 50.5 μM) (Figures 6B and 6C). Cytotoxicity assessment showed that aptE-Fc/3-2_32 did not exhibit marked cytotoxicity relative to the control aptamer aptE-Fc/3-1_37 (Figure S3). These results indicated that aptE-Fc/3-2_32 retained the ability to inhibit TBEV infection at a level comparable to that of the parental aptamer aptE-Fc/3-2.

## Discussion

Aptamers have potential advantages over antibodies in virology, including improved stability and reproducibility. However, despite extensive experimental efforts, aptamer-based reagents have not been widely adopted in practical or clinical applications for the treatment or diagnosis of viral infections. In this study, we identified novel TBEV-specific ssDNA aptamers, characterized their biochemical and functional properties, and evaluated their potential for advanced diagnostic and antiviral applications.

Previous studies on anti-TBEV aptamers have mainly evaluated binding to recombinant E protein fragments or isolated antigens, whose relationship to the antigenic properties of native viral particles has not been fully characterized.^10^^,^^11^ Consequently, little is known about the extent to which aptamers recognize the antigenic structures present in TBEV particles. In this study, four major aptamer candidates were identified by SELEX using recombinant TBEV E-Fc as the target molecule (Figures 1 and 2A), and their binding to the TBEV E protein was confirmed (Figure 2D). Among these candidates, aptE-Fc/3-1 and aptE-Fc/3-2 showed high binding affinities to SPs derived from two TBEV strains (Figures 2B and 2C), which are composed of prM and E proteins, and retain the structural and antigenic features of TBEV particles.^14^^,^^15^ These results indicated that the identified aptamers recognized the antigenic structures present on the TBEV particles. In addition, the binding affinities of aptE-Fc/3-1 and aptE-Fc/3-2 were comparable to those of previously reported anti-TBEV aptamers, including TBEV-2F, TBEV-2R, and TBEV-8R^10^ (Figure 2C), indicating that the newly identified aptamers possess properties suitable for further application-oriented analyses.

To further explore their diagnostic potential, we evaluated whether the aptamers could be incorporated into *in vitro* TBEV detection systems. Aptamers aptE-Fc/3-1 and aptE-Fc/3-2 enabled the detection of immobilized TBEV antigens (Figure 4A). In addition, combinations of the capture and probe aptamers generated detectable signals (Figure 4B). These results supported the use of these aptamers as antibody-independent diagnostic platforms. Given the practical advantages of aptamers over antibodies,^1^^,^^3^ our findings provide a basis for the development of aptamer-based diagnostic systems that target TBEV.

In general, shortening aptamer sequences may contribute to reducing synthesis costs and improving practical applicability.^23^^,^^24^ Both aptE-Fc/3-1_37 and aptE-Fc/3-2_32 retained binding and detection capabilities comparable to or better than those of their parent aptamers (Figures 3 and 4). These findings indicate that the truncated aptamers preserve the functional properties required for the recognition of TBEV particles and may therefore be applicable as TBEV-targeting molecular tools, analogously to the original aptamers.

As a preliminary evaluation of the potential for *in vivo* applications, the stability of the aptamers in serum and their reactivity with TBEV-infected cells were examined. The aptamers aptE-Fc/3-1 and aptE-Fc/3-2 retained their binding activity for several hours in the presence of serum (Figure 5A), suggesting acceptable stability under *in vivo*-like conditions. In addition, higher fluorescence signals were detected in TBEV-infected cells than in uninfected cells following incubation with fluorophore-labeled aptamers (Figure 5B). These findings indicated that the aptamers obtained in this study possessed basic properties for *in vivo* applications, including biological stability and target recognition. Further studies using TBEV-infected animal models are important to evaluate their biodistribution, clearance and *in vivo* targeting properties.

Interestingly, the truncated variant aptE-Fc/3-2_32, identified during characterization of the newly obtained aptamers, maintained its binding activity in the presence of serum for a longer period than the full-length sequence (Figure 5A). Enhanced structural stability and nuclease resistance have similarly been observed upon the removal of non-essential sequence regions in previous studies.^25^^,^^26^ Our data suggest that sequence truncation can improve the biological stability of TBEV-targeting aptamers, without compromising their ability to recognize viral particles, thereby increasing their potential utility for future *in vivo* applications.

Several aptamers exhibited inhibitory activity against TBEV infection *in vitro*, and both aptE-Fc/3-2 and its truncated variant aptE-Fc/3-2_32 showed concentration-dependent antiviral effects (Figure 6). In contrast, none of the aptamers inhibited JEV infection. Taken together with the selective binding to the TBEV E protein (Figure 2D), these results suggest that the antiviral activity of these aptamers is associated with their specific recognition of the TBEV E protein. Notably, aptE-Fc/3-2 also demonstrated partial inhibitory effects against LGTV, a member of the TBEV serocomplex distinct from JEV (Figure 6A). These findings suggest that some aptamers recognize antigenic features conserved among TBEV-related flaviviruses and may therefore have potential as broad-spectrum anti-TBEV molecules. However, the mechanisms underlying the antiviral activity of these aptamers remain unclear. Further structural and functional characterization of the interaction between the aptamer and E protein, including the binding site, E protein occupancy on viral particles, interactions with host-cell receptors, and stability of the aptamer-E protein interaction under physiological conditions, will be important for elucidating the mechanisms of antiviral action. Although the antiviral effects were modest, optimization of aptamer sequences may also facilitate the rational design of aptamers with improved antiviral efficacy.

TBEV infection is characterized by viral replication in the CNS, where efficient delivery of therapeutic agents remains a major challenge.^8^^,^^27^ In this study, we identified several ssDNA aptamers against TBEV, some of which exhibited reactivity to infected cells and retained binding activity under serum conditions (Figure 5), indicating their potential as targeting molecules for selective localization to virus-infected sites. In addition to these properties, several aptamers exhibited inhibitory activity against TBEV infection *in vitro* (Figure 6). These findings provide a basis for the development of aptamer-based platforms for targeted delivery strategies against TBEV infection, and may further support theranostic applications that integrate targeted delivery and functional intervention. In future studies, chemical modification of the aptamers identified in this study, including 3′-end protection, may provide an approach to improve their *in vivo* stability while preserving their binding and antiviral activities. Assessment of their *in vivo* performance and pharmacokinetic properties, including their ability to cross the blood-brain barrier and selectively target virus-infected cells in the CNS, will also be important to determine the feasibility of these aptamers for targeted delivery.

In conclusion, we have identified novel ssDNA aptamers that recognize TBEV particles, viral antigens, and infected cells. The identified aptamers were applicable to diagnostic assays and exhibited biological properties relevant to antiviral and targeting applications. These findings provide a foundation for future studies aimed at developing aptamer-based diagnostic, therapeutic, and theranostic strategies targeting TBEV and related flaviviruses.

## Materials and methods

### Cells and viruses

Baby hamster kidney fibroblast (BHK) cell line was cultured at 37°C in Eagle’s minimum essential medium (EMEM; Fujifilm Wako Pure Chemical Corporation, Osaka, Japan) containing 8% (v/v) fetal bovine serum (FBS) and penicillin/streptomycin. Huh7 cells were cultured at 37°C in D-MEM (high glucose) with L-glutamine and phenol red (Fujifilm Wako Pure Chemical Corporation) containing 10% (v/v) FBS and penicillin/streptomycin. Expi293F cells were cultured in Expi293 Expression Medium (Thermo Fisher Scientific, Waltham, MA, USA) at 37°C with 8% CO_2_ and shaking at 125 rpm.

The viruses used in this study included two strains of TBEV (Oshima 5-10 and Sofjin), one strain of LGTV (TP21), a member of the TBEV serocomplex, and one strain of JEV (JaOArS982). The amino acid sequence homology of E proteins among the viral strains used in this study is shown in Table S1. Each virus strain was propagated in BHK cells and then stored as a stock virus at −80°C. The virus titers (PFU ml^−1^) were determined by a plaque assay in BHK cells, as described previously.^9^ All TBEV infection experiments were performed at the Biosafety Level-3 laboratory at Nagasaki University, Japan, according to the institutional guidelines.

### Production of recombinant proteins

For the preparation of recombinant viral envelope proteins, we used the Expi293 Expression System (Thermo Fisher Scientific) according to the manufacturer’s instructions. Briefly, Expi293F cells were transfected with pCAGGS-based plasmids encoding prM and full-length E proteins of the Oshima 5-10 or Sofjin strain to produce secreted SPs^14^^,^^15^ or with plasmids encoding prM and E in which the C-terminal transmembrane region was replaced with the Fc region of rabbit IgG (E-Fc).^13^ Secreted E-Fc was purified using an Ab-Capcher ExTra (ProteNova, Kagawa, Japan).

### SDS-PAGE and western blotting

The production and purification of recombinant proteins were confirmed by SDS-PAGE and immunoblotting (Figure S4). Briefly, samples were resolved on commercial 5%–20% gradient polyacrylamide gels (ATTO, Tokyo, Japan) and stained with Coomassie Brilliant Blue (CBB) (ATTO). For immunoblotting, separated proteins were transferred onto polyvinylidene difluoride (PVDF) membranes (Merck, Darmstadt, Germany) and blocked with Block Ace (KAC, Kyoto, Japan). The membranes were incubated with anti-TBEV E antibodies^28^ for 1 h, followed by incubation with horseradish peroxidase (HRP)-labeled anti-mouse IgG antibodies (1:1,000). For E-Fc detection, we used an HRP-labeled anti-rabbit IgG antibody (1:3,000). Proteins were visualized using a chemiluminescent HRP substrate (Merck) and a LuminoGraph I (ATTO) imaging system.

### ssDNA library and primers

The initial ssDNA aptamer library used in this study contained a 40-base randomized region in the center flanked by primer hybridization regions, yielding an 81-nucleotide (nt) total length (Figure 1A): 5′-AGTATACGTATTACCTGCAGC-(N)_40_-CGATATCTCGGAGATCTTGC-3′).^16^ The primer sequences used for PCR amplification were 5′-AGTATACGTATTACCTGCAGC-3′ (forward primer) and 5′-GCAAGATCTCCGAGATATCG-3′ (reverse primer). The ssDNA library, primers, and aptamers were synthesized by Integrated DNA Technologies, Inc. (Coralville, IA, USA).

### Aptamer selection

Aptamers were selected by systematic evolution of ligands by exponential enrichment (SELEX). An outline of the ssDNA aptamer selection performed in this study is shown in Figure 1B. The initial ssDNA library was dissolved in binding buffer (DPBS with 100 mg/L CaCl_2_ and 100 mg/L MgCl_2_ [Gibco, Thermo Fisher Scientific] containing 0.05% Tween 20 [Thermo Fisher Scientific]) and denatured by heating at 95°C for 5 min and immediately cooled on ice for 5 min to allow intramolecular folding. Folded ssDNA was mixed with 50 μL of Dynabeads Protein G (VERITAS, Tokyo, Japan) and rotated at room temperature for 30 min as a counter-SELEX step. A solution containing unbound ssDNA was collected using a magnetic stand. The recovered ssDNA was then incubated with E-Fc derived from the Oshima 5-10 strain immobilized on magnetic beads (Figure 1C) with rotation at room temperature to perform the SELEX step. Aptamer-bound E-Fc was recovered by magnetic capture, washed multiple times with 300 μL of PBST, and resuspended in 50 μL of nuclease-free water. The selection stringency at each step was progressively enhanced to select aptamers that specifically recognized E-Fc (Table S2).

To obtain sufficient ssDNA for the next round of aptamer selection, a two-step PCR amplification followed by purification was performed.^29^ First, symmetric PCR was performed using the ssDNA resuspension solution recovered after selection with E-Fc-bound beads as a template. The symmetric PCR was performed in a total volume of 50 μL, consisting of 10 μL of template, 10 μL of 5× Colorless GoTaq Reaction Buffer (Promega, Madison, WI, USA), 1 μL of 10 mM PCR Nucleotide Mix (Promega), 0.25 μL of GoTaq DNA polymerase (Promega), and 50 pmol of each 5′-biotinylated forward and reverse primers. The PCR was performed under the following conditions: an initial denaturation at 95°C for 5 min, followed by 11 cycles of 95°C for 1 min, 55°C for 1 min, and 72°C for 1 min, and a final extension at 72°C for 5 min. Next, asymmetric PCR was performed in a 20 μL reaction volume, consisting of 1 μL symmetric PCR product, 20 pmol of non-biotinylated forward primer, 1 pmol of 5′-biotinylated reverse primer, and 10 μL of 2× PrimeSTAR Max (Takara Bio Inc., Shiga, Japan). The PCR was performed under the following conditions: an initial denaturation at 98°C for 5 min, followed by 30 cycles of 98°C for 10 s, 55°C for 15 s, and 72°C for 5 s, and a final extension at 72°C for 2 min. Finally, the asymmetric PCR products were mixed with Streptavidin MagneSphere Paramagnetic Particles (Promega) for 20 min at room temperature, and the target ssDNA was recovered by removing the biotinylated dsDNA using a magnetic stand. The ssDNA solution was then used for the next round of aptamer selection.

### Sequencing and secondary structure analysis

After the seventh round of aptamer selection, the collected ssDNA aptamers were amplified by conventional PCR and cloned into a TOPO vector (Invitrogen, Carlsbad, CA, USA) according to the manufacturer’s instructions. The inserted DNA from each clone was sequenced using a BigDye Terminator v3.1 Cycle Sequencing Kit (Applied Biosystems, Foster City, CA, USA) on a 3500 Genetic Analyzer (Applied Biosystems). To classify the clones, a phylogenetic analysis of the obtained sequences was performed using MEGA11.^30^ The secondary structure of the aptamers was predicted using the RNAfold web server RNAfold (http://rna.tbi.univie.ac.at/cgi-bin/RNAWebSuite/RNAfold.cgi).

### Evaluation of aptamer binding to target proteins

The binding affinity and specificity of each aptamer were evaluated using target-coated microplates. Briefly, TBEV SPs or E proteins from Triton X-100-treated TBEV were captured using an anti-TBEV E monoclonal antibody 19/1786^31^ on microplates, and E-Fc was captured using an anti-rabbit IgG antibody. As a negative antigen control, PBST supplemented with 0.5% bovine serum albumin (BSA) was added instead of the target antigen. Microplates were blocked and washed using Block Ace and PBST, respectively, as described previously.^27^ The captured target proteins were incubated with the respective 5′-FAM- or 5′-biotin-labeled aptamer at 37°C for 60 min, and then washed with PBST. The fluorescence intensity of FAM (excitation 485 nm, emission 535 nm) was measured using a plate reader SpectraMax iD5 plate reader (Molecular Devices, Sunnyvale, CA, USA). The 5′-biotin-labeled aptamer was reacted with HRP-conjugated streptavidin (Jackson ImmunoResearch Laboratories Inc., West Grove, PA, USA), followed by color development using *o*-phenylenediamine dihydrochloride (OPD) (Thermo Fisher Scientific). After 30 min of incubation, the OD was measured at 450 nm using the SpectraMax iD5.

An aptamer-based sandwich assay was performed to evaluate whether the selected aptamers were suitable for capturing the target proteins. Briefly, 50 μL of 500 nM 5′-biotin-labeled aptamer (capture aptamer) was coated on a microplate using streptavidin and incubated at 4°C overnight. Target protein addition, blocking, and washing steps were performed as described previously. Next, 500 nM of 5′-FAM-labeled aptamer (probe aptamer) was incubated at 37°C for 60 min. The fluorescence intensity was measured as described previously.

### SPR analysis

The binding affinity of the truncated aptamer for E-Fc was evaluated using SPR. SPR measurements were performed at 25°C using BiacoreX100 (Cytiva, Marlborough, MA, USA). The 5′-biotinylated polydT (16 mer) was immobilized on the surface of a sensor chip SA (Cytiva, Marlborough, MA, USA) to Flow cell 2 as a ligand at a level of approximately 300–500 Resonance units (RUs). After immobilization, the sensor chip was washed and equilibrated with SPR buffer (10 mM sodium phosphate (pH 7.0), 140 mM NaCl, 2.6 mM KCl, 0.5 mM MgCl_2_, 0.9 mM CaCl_2_, and 0.05% Tween 20). The 3′-polyA (16 mer)-tagged aptamer dissolved in SPR buffer was immobilized to approximately 30 RUs in Flow cell 2. Subsequent analyses were performed using a multi-cycle kinetics method at 30 μL/min. The association time was 180 s, and the dissociation time was 900 s. TBEV E-Fc in SPR buffer was injected as an analyte at concentrations of 38, 75, 150, 300, and 600 nM or 75, 150, 300, 600, and 1,200 nM. The concentration range of each analyte was optimized according to the expected binding affinity to obtain reliable kinetic fitting. The regeneration step was performed after dissociation. The sensor chip surface was regenerated for 60 s with regeneration solution (1M NaCl, 50 mM NaOH) at a flow rate of 10 μL/min. After regeneration, the response returned to baseline, confirming baseline stability. Blank buffer injections were performed prior to analyte cycles for double-referencing. Sensorgrams were analyzed using the BIA evaluation software (Cytiva, Marlborough, MA, USA). A Langmuir (1:1) binding model was used to analyze the association rate constant, *k*_on_ (M^−1^s^−1^), the dissociation rate constant, *k*_off_ (s^−1^), and the dissociation constant, *K*_d_ (M).

### NMR spectroscopy

To confirm G-quadruplex formation in the aptamer predicted to form a G-quadruplex based on secondary structure prediction, we measured the NMR spectra. The sample was annealed by heating at 95°C for 5 min followed by snap cooling on ice. The sample was dissolved in 20 mM sodium phosphate (pH 6.5) or 20 mM potassium phosphate (pH 6.5). Concentrations of the sample was 0.2 mM. NMR spectra were measured using an AVANCE *NEO* 600 spectrometer (Bruker BioSpin). Spectra were recorded at a probe temperature of 10°C using the jump-and-return schemes^32^ for water suppression.

### Biostability assay

To assess biostability under serum conditions, the 5′-biotin-modified aptamer solution (100–500 nM) was incubated at 37°C in 50% mouse serum (obtained from BALB/c mice [Japan SLC, Inc., Shizuoka, Japan], without heat inactivation) as described previously.^33^^,^^34^ Aptamer mixtures recovered at different time points were heated at 90°C for 10 min and stored at −20°C until use. Residual aptamers were detected by incubating the recovered mixtures with SPs captured on microplates, followed by detection using streptavidin-conjugated HRP, as described previously. Corrected values were calculated by subtracting the values of the control wells at each recovery time point. Changes in binding affinity were determined using the corrected value immediately after mixing with mouse serum (0 h) as a base line.

### Evaluation of aptamer binding to TBEV-infected cells

The binding affinity of each aptamer to TBEV-infected cells was evaluated by measuring fluorescence intensity. BHK cells cultured to 90% confluence in a tissue culture-treated 96-well plate (Revvity, Waltham, MA, USA) were infected with the Oshima 5-10 strain at a multiplicity of infection (MOI) of 0.1. At 2 days post-infection, the cells were washed three times with PBS, followed by incubation with 400 nM 5′-Alexa Flour® 647-labeled aptamers at 37°C. After incubation for 1 h, the cells were washed thrice with PBS and fixed with 3.7% formalin for 1 h. Fluorescence intensity (excitation 640 nm/emission 690 nm) was measured using a SpectraMax iD5.

### Inhibition of viral infection by aptamer

The inhibition of viral infection by each aptamer was evaluated using a modified PRNT. Briefly, aptamers were mixed with 100 PFU of virus and incubated at 37°C for 60 min. The reaction mixture was inoculated into BHK cells in 12-well plates, and the number of plaques was counted 3–4 days post-infection. The infection inhibition rate for each aptamer was calculated based on plaque counts in the negative control (PBS only). Statistical comparisons of the infection inhibition rates were performed using random ssDNA controls (the initial ssDNA aptamer library). Cell viability 24 h after aptamer treatment was assessed using a CCK-8 assay (Cell Counting Kit-8, Dojindo laboratories, Kumamoto, Japan), according to the manufacturer’s instructions.

### Statistical analysis

GraphPad Prism (version 9.5.1, GraphPad Software Inc., San Diego, CA, USA) was used for all statistical analyses in this study. All experiments were performed in three independent replicates, and data are expressed as mean ± standard deviation (SD).

## Data and code availability

The data supporting the findings of this study are available within the article and its supplemental information. Additional data are available from the corresponding author upon reasonable request.

## Acknowledgments

This work was supported by Grants-in-Aid for Scientific Research (21KK0123, 23K27079, 23K19331, and 24K18022) from the Ministry of Education, Culture, Sports, Sciences, and Technology of Japan, 10.13039/100009619AMED (JP25fm0208101, JP25wm0325059, and JP223fa627004), and the 10.13039/100007449Takeda Science Foundation. This work was partly supported by the Cooperative Research Project Program of the National Research Center for the Control and Prevention of Infectious Diseases at 10.13039/100015060Nagasaki University.

## Author contributions

Y.F.: conceptualization, investigation, formal analysis, data curation, visualization, funding acquisition, writing – original draft, writing – review and editing; T.S.: investigation, writing – review and editing; M.H.: methodology, Resource, writing – review and editing; M.F.: methodology, Resource, writing – review and editing; T.S.: investigation, methodology, funding acquisition, writing – review and editing; K.Y.: project administration, funding acquisition, writing – review and editing.

## Declaration of interests

The authors declare no conflict of interest.

## Supplemental information

Document S1. Figures S1–S4 and Tables S1 and S2

Document S2. Article plus supplemental information
