# Supplementary material for: Selection and characterization of ssDNA aptamers targeting tick-borne encephalitis virus particles
Source: Mol Ther Nucleic Acids. 2026 Oct 12;37(4):103138. doi: 10.1016/j.omtn.2026.103138 (PMC13734832; doi:10.1016/j.omtn.2026.103138)
Supplement: Supplementary file 1 [file mmc1.pdf]

OMTN, Volume *37*

Supplemental information

Selection and characterization of ssDNA aptamers targeting tick-borne encephalitis virus particles

Yuji Fujii, Tomohiro Shinozaki, Minato Hirano, Mizuki Fukuta, Taiichi Sakamoto, and Kentaro Yoshii

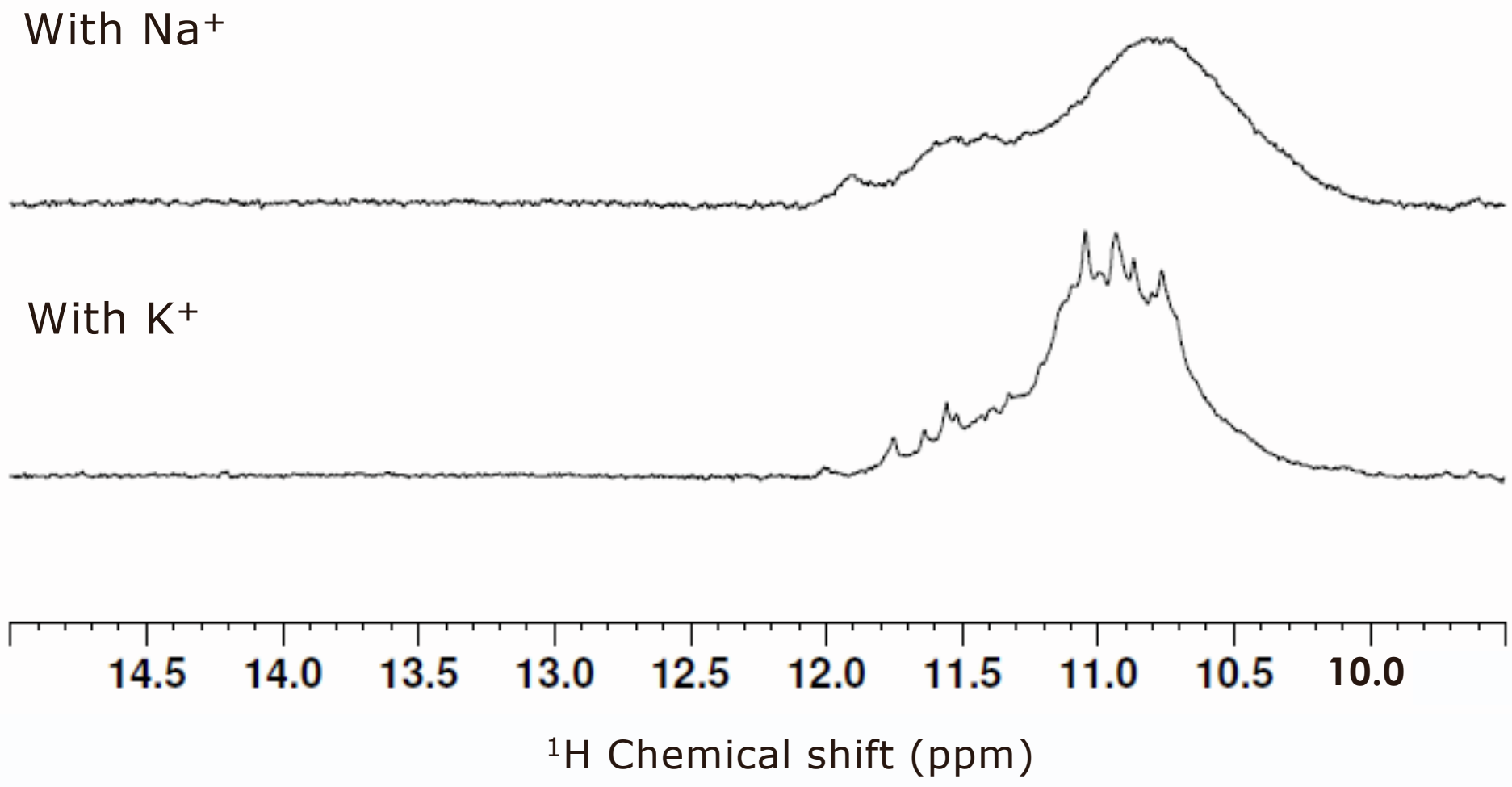

Figure S1. NMR spectra of aptE-Fc/3-2_32.
NMR spectra of aptE-Fc/3-2_32 were measured in the presence of Na^+ or K^+. Imino proton signals characteristic of G-quadruplex structures were observed in the 10–12 ppm region. The signals were broad in the presence of Na^+, whereas some sharp signals were observed in the presence of K^+.

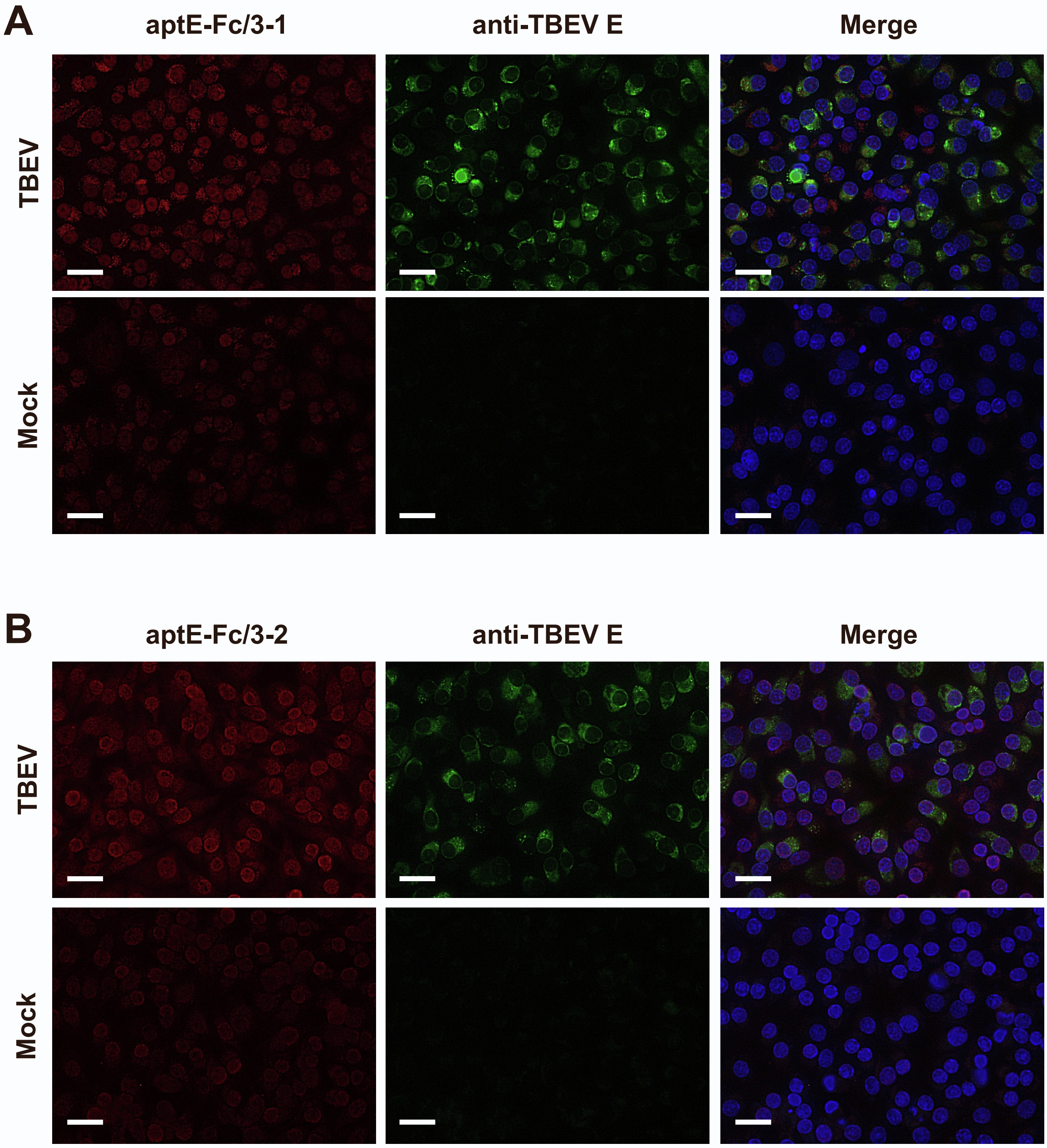

Figure S2. Fluorescence microscopy images of TBEV-infected cells stained with aptamers. Representative fluorescence microscopy images of TBEV-infected and mock-infected cells stained with Alexa Fluor® 647-labeled aptE-Fc/3-1 (A) and aptE-Fc/3-2 (B). TBEV E protein was detected using antibody 19/1786 and visualized in green. Images were acquired using a BZ-X800 fluorescence microscope (KEYENCE Corporation, Osaka, Japan). Scale bars, 50 µm.
TBEV, tick-borne encephalitis virus.

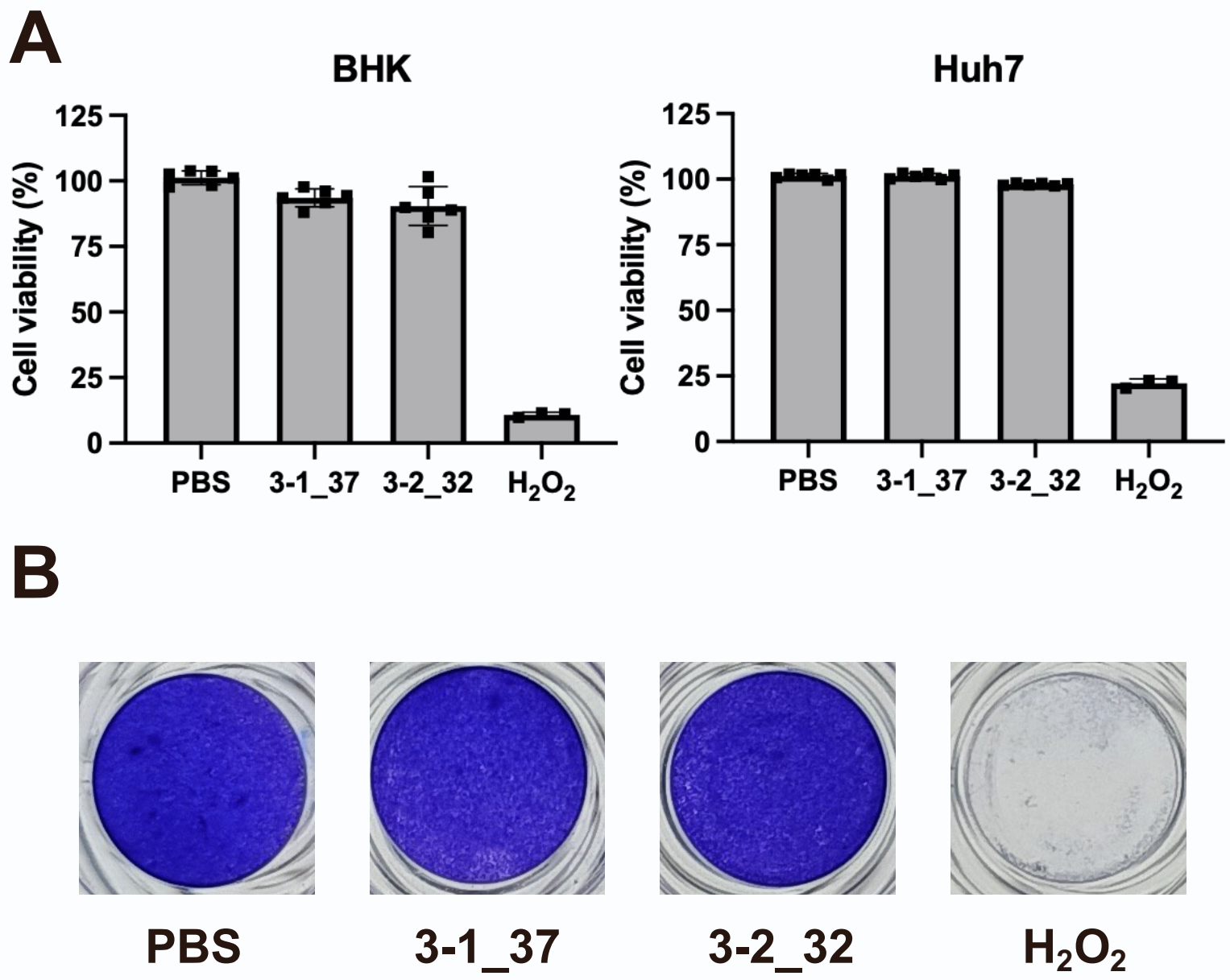

Figure S3. Cytotoxicity of aptamers.
(A) Cytotoxicity was assessed in BHK and Huh7 cells using a CCK-8 assay after treatment with aptamers at 20 μM. Hydrogen peroxide (H_2O_2; final concentration, 3%) was used as a positive control for cytotoxicity. Cell viability was normalized to that of untreated cells. (B) Representative crystal violet-stained images of BHK cells treated with aptamers at 20 μM are shown.

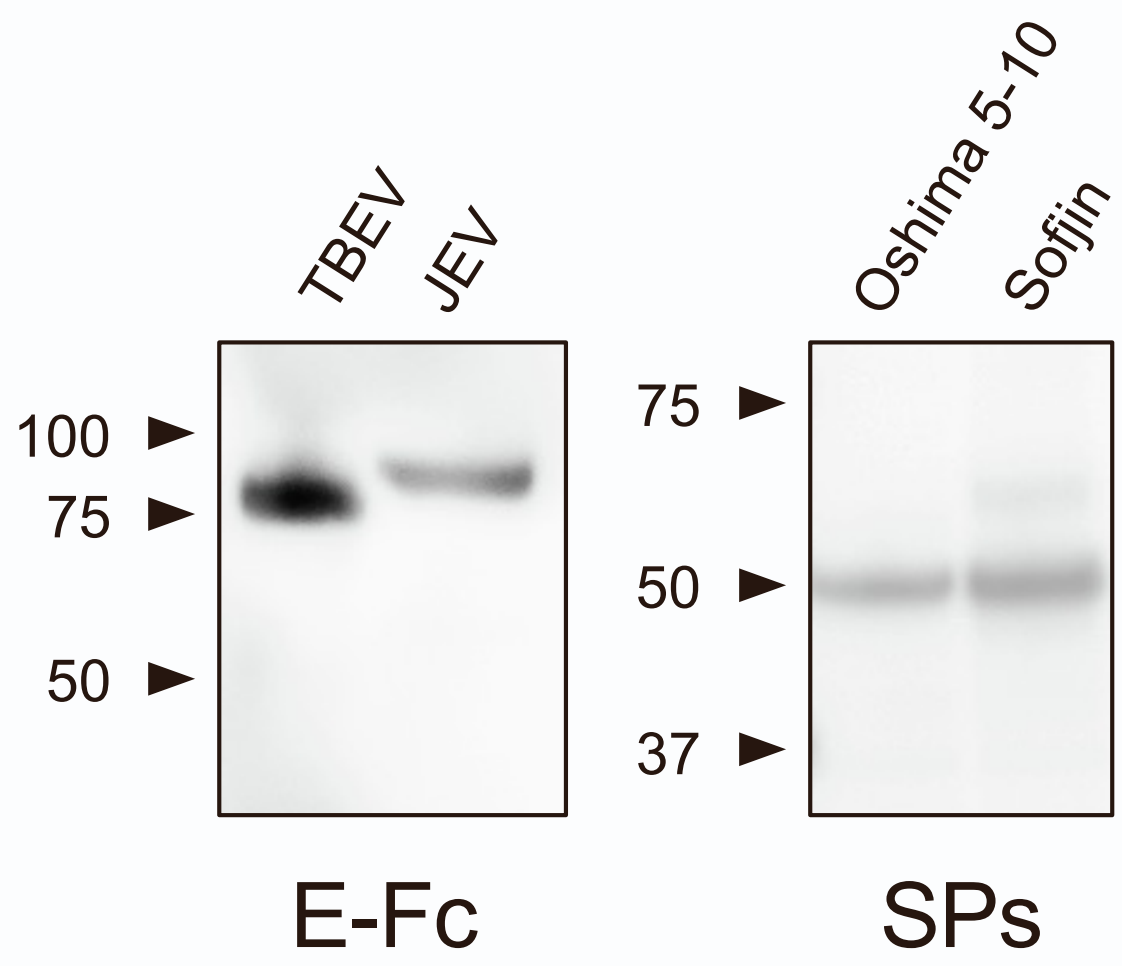

Figure S4. Western blot analysis of E-Fc and subviral particles (SPs).
Expression of E-Fc and SPs was confirmed by Western blotting. Numbers indicate molecular weight markers (kDa).
TBEV, tick-borne encephalitis virus; JEV, Japanese encephalitis virus.

Table S1. Comparison of E protein amino acid identities among the viral strains used in this study.

	TBEV_Oshima_5-10	TBEV_Sofjin	LGTV_TP21	JEV_JaOArS982
TBEV_Oshima_5-10				
TBEV_Sofjin	99.2%			
LGTV_TP21	88.8%	88.4%		
JEV_JaOArS982	41.4%	41.6%	41.4%	

Amino acid identities were calculated from pairwise amino acid p-distances using MEGA12 software. Sequence data were retrieved from GenBank. The accession numbers for strains Oshima 5-10, Sofjin, TP21, and JaOArS982 are AB062063, JX498940, NC_003690, and M18370, respectively.

Abbreviations: TBEV, tick-borne encephalitis virus; LGTV, Langat virus; JEV, Japanese encephalitis virus.

Table S2. The stringency of conditions of aptamer selection.

Round	Amount of newly supplied library (pmol)	SELEX time (min)	Target volume (μL)	Number of washes
1	2,500	30	30	3
2	500	30	30	3
3	250	30	30	3
4	0	30	30	3
5	0	30	10	3
6	0	10	10	5
7	0	10	3	5

Abbreviation: SELEX, systematic evolution of ligands by exponential enrichment.
